# Deaths from COVID-19 in healthcare workers in Italy—What can we learn?

**DOI:** 10.1017/ice.2020.241

**Published:** 2020-05-15

**Authors:** Pierfrancesco Lapolla, Andrea Mingoli, Regent Lee

**Affiliations:** 1 Department of Surgery P. Valdoni, Policlinico Umberto I, Sapienza University of Rome, Rome, Italy; 2 Nuffield Department of Surgical Sciences, University of Oxford, Oxford, United Kingdom

*To the Editor*—The novel coronavirus disease (COVID-19) pandemic is imposing a significant burden on healthcare systems worldwide. On April 16, 2020, the Italian National Institute of Health (ISS) reported that 16,991 healthcare workers (HCWs) had tested positive for severe acute respiratory syndrome coronavirus 2 (SARS-CoV-2). These HCWs had a median age of 48 years, and 68% were female and 32% were male, which is in line with the ratio in the Italian healthcare system (66.8% female and 33.2% male).^1,2^ The infected HCWs accounted for 10.7% of the total number of positive cases (n = 168,941).^1^

Since the first case in the outbreak on February 21, the number of HCW deaths has risen dramatically. On April 17 the latest estimate of medical doctor deaths reached 119, which is 57.8% of total HCW deaths; followed by nurses 16.5% (n = 34), nurse aides 8.3% (n = 17) and dentists 5.8% (n = 12) (Fig. 1).^3^ The COVID-19–related deaths include 2 nurses who committed suicide due to unsustainable pressure at work.^4^ No other country has seen the same elevated number of doctor deaths; China, where the epidemic began in December, had fewer.^5^ General practitioners seem to be the worst hit among all medical specialties, registering 32% deaths (n = 66) (Fig. 1).^3^ This high rate could reflect their presence in the first line of defense for anyone presenting with the initial symptoms.

**Fig. 1.**
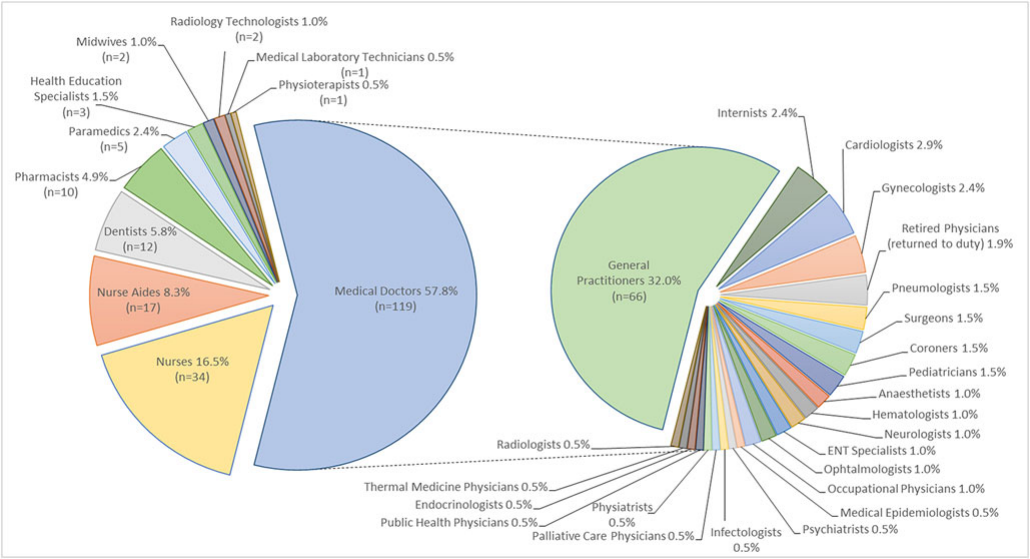
Healthcare worker deaths from COVID-19 outbreak in Italy by category and medical specialty.*

* Latest data are from the Italian National Federation of Orders of Surgeons and Dentists (FNOMCeO), the National Federation of Professional Nursing Orders (FNOPI), Italian Federation of Pharmacists (FOFI), National Federation of Medical Radiology Technicians, Technical Health Professions, Rehabilitation and Prevention Orders (FNO-TSRM-PSTRP), National Federation of Health and Social Health Professions (MIGEP). Data accessed April 17 2020.

Data available from the ISS on confirmed cases and deaths by age distribution indicate that 34% of the total HCWs testing positive (n = 16,953) were aged 50–59 years and that 28.2% were aged 40–49 years. Of HCW deaths, 43.3% were aged 60–69 years and 26.7% were aged 50–59 years. HCWs aged 70–79 years comprised 20% of HCW deaths (12.6% case fatality rate).^1^

On April 9, 2020, the ISS ran a retrospective epidemiological analysis of the number of infected HCWs by category, care context, and site where the infection presumably occurred, together with type of activity carried out at the time of infection. These data are available for 16,179 of the 16,991 HCWs confirmed positive for the virus. Nurses and midwives together are the most represented with 43.2% (n = 6,988) of all infected HCWs, followed by doctors 22% (n = 3,574) divided between hospital doctors 19% (n = 3,071), general practitioners 0.8% (n = 130) and other doctors 2.3% (n = 373).^1^ Data for the healthcare context in which the infections presumably occurred are available for 11,738 HCWs; of these, 70.9% have contracted COVID-19 while serving in hospitals or in emergency care services (ambulance assistance).^1^

Interestingly, according to the National Federation of Orders of Surgeons and Dentists (FNOMCeO) registry,^2^ general practitioners accounted for the highest number of HCW deaths (Fig. 1) despite being the least infected group (as reported in the latest ISS analysis).^1^ Furthermore, according to the National Federation of Professional Nursing Orders (FNOPI), 32% of the nurse deaths by April 16, 2020, initially contracted the virus while on duty in nursing care homes where personal protective equipment (PPE) was mostly lacking, and 50% were working in nonhospital healthcare facilities.^4^

The sheer intensity of the COVID-19 outbreak in Italy, the recruitment of elderly retired doctors and shortages of PPE, particularly in nonhospital care, might be among relevant factors contributing to the elevated number of fatalities among HCWs in this country. Therefore, it is essential to carry out another retrospective epidemiological investigation and a prospective study to identify the main risk factors contributing to COVID-19–related deaths in the different HCWs categories in order to produce viable schemes for their protection. At this point, what lesson can other countries learn from the Italian sacrifice? Protecting and testing HCWs must be a top priority; governments will not be forgiven for needless deaths.

## References

[1] COVID-19 integrated surveillance: key national data. COVID-19 epidemic. 16 April 2020 national update. (In Italian). Italian National Institute of Health (ISS) website. https://www.epicentro.iss.it/coronavirus/sars-cov-2-sorveglianza-dati. Published April 16, 2020. Accessed April 17, 2020.

[2] Italian Ministry of Health. National healthcare staff, 2017 data [in Italian]. Italian Ministry of Health website. http://www.salute.gov.it/portale/news/p3_2_1_1_1.jsp?lingua=italiano&menu=notizie&p=dalministero&id=3845. Published July 31, 2019. Accessed April 17, 2020.

[3] Registry of doctor deaths during COVID-19 epidemic [in Italian]. Italian National Federation of Orders of Surgeons and Dentists (FNOMCeO) website. https://portale.fnomceo.it/elenco-dei-medici-caduti-nel-corso-dellepidemia-di-covid-19/. Updated May 15, 2020. Accessed May 15, 2020.

[4] National Federation of Professional Nursing Orders (FNOPI) website [in Italian]. https://www.fnopi.it/ Accessed April 17, 2020.

[5] Zhan M , Qin Y , Xue X , et al. Death from Covid-19 of 23 healthcare workers in China. N Engl J Med 2020 Apr 15. doi: 10.1056/NEJMc2005696. PMC7179960 32294342

